# Supplementary material for: Ferritin Mitochondrial (FTMT)-Driven Mitochondrial Ferroptosis in Vascular Smooth Muscle Cells: A Role of NCOA4 in Atherosclerosis Pathogenesis and Modulation by Gualou–Xiebai
Source: Nutrients. 2025 Nov 26;17(23):3713. doi: 10.3390/nu17233713 (PMC12694305; doi:10.3390/nu17233713)
Supplement: Supplementary file 1 [file nutrients-17-03713-s001.zip › nutrients-3969812-supplementary.pdf]

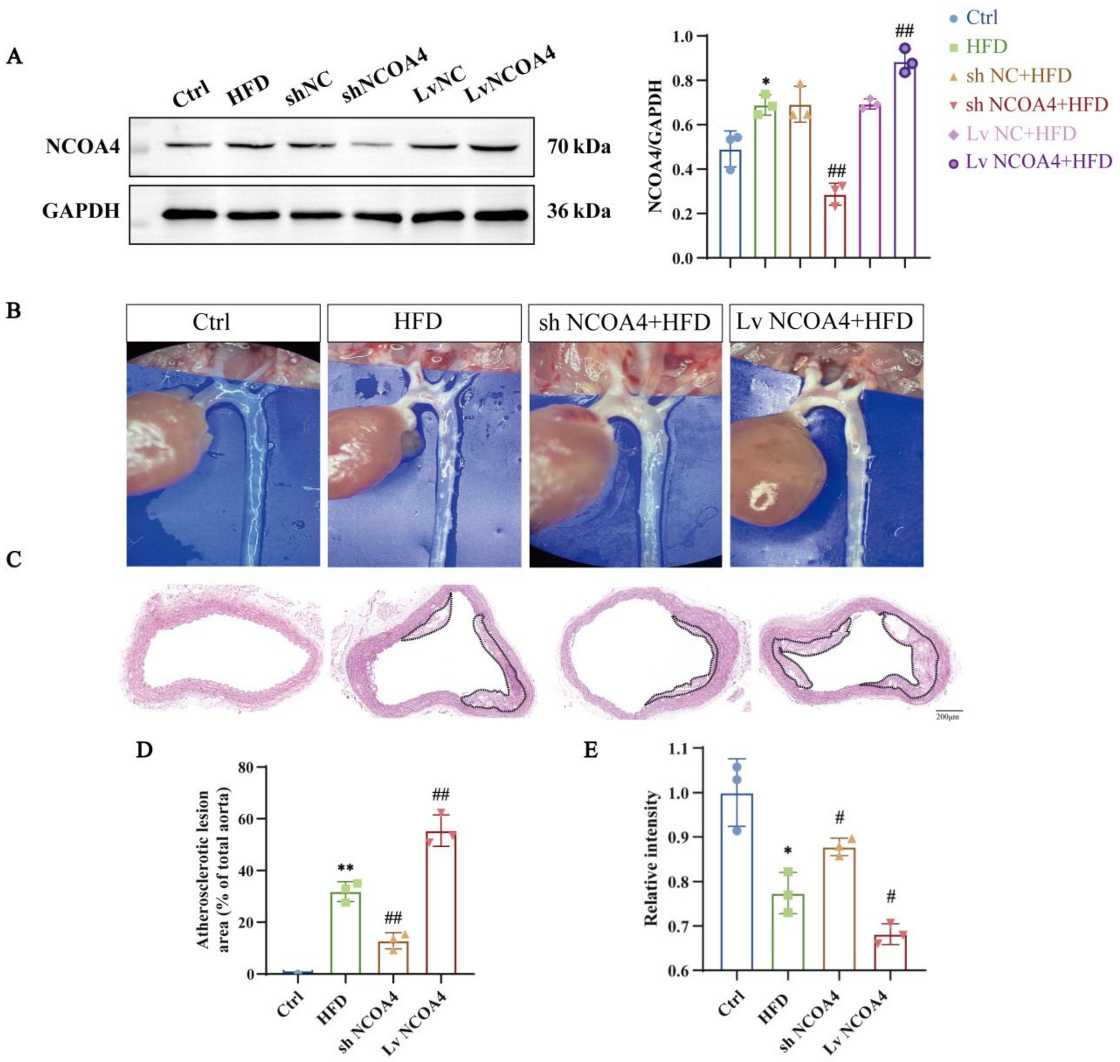

Supplementary Figure S1. NCOA4-mediated ferroptosis inhibits the proliferation and migration of VSMCs. (A) The validation of NCOA4 lentiviral transfection efficiency (n=3). (B) Aortic imaging showing aortic lipid deposition in each group. (C,D) HE staining of aortic in each group (scale bar: 200μm, D: quantitative analysis). (E) Quantitative analysis of the co-localization of α-SMA (red)and GPX4 (green) in atherosclerotic lesions. $^{*}P < 0.05$, $^{**}P < 0.01$ vs. control group; $^{\#}P < 0.05$, $^{\#\#}P < 0.01$ vs. HFD group.

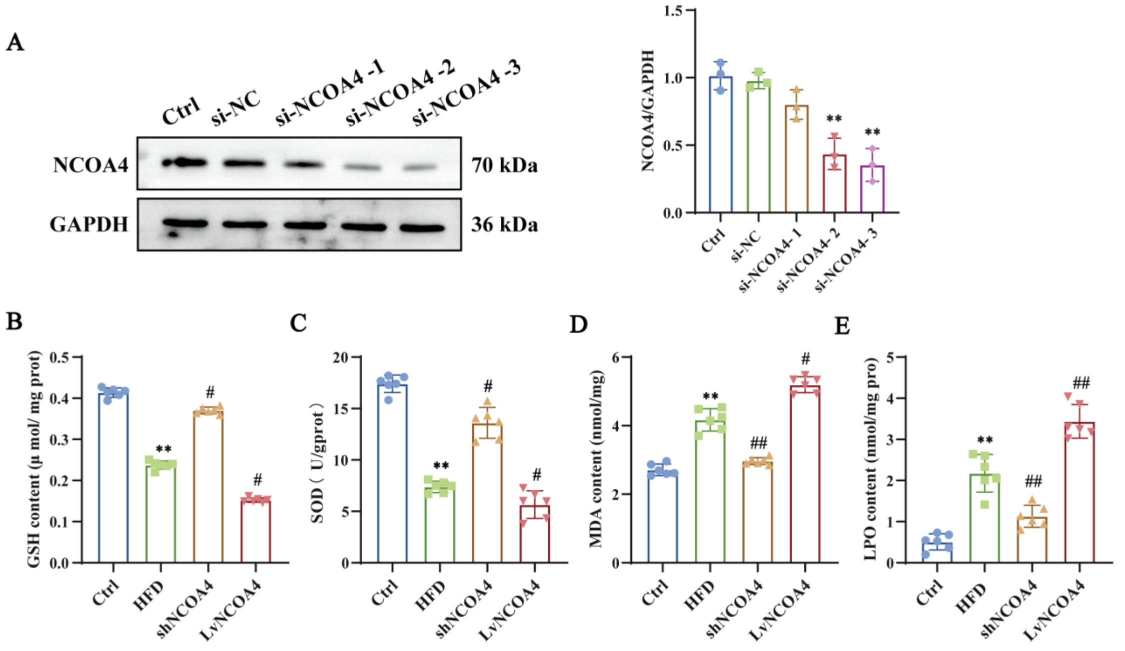

Supplementary Figure S2. Silencing NCOA4 reduces ox-LDL-induced VSMCs proliferation and migration by suppressing ferroptosis. (A) Western blot to detect the transfection efficiency of silencing NCOA4 in VSMCs (n=3). (B-E) Biochemical kit for GSH, SOD MDA and Fe levels (n =6). $^{**}P < 0.01$ vs. si-NC group, $^{\#}P < 0.05$, $^{\#\#}P < 0.01$ vs. HFD group.

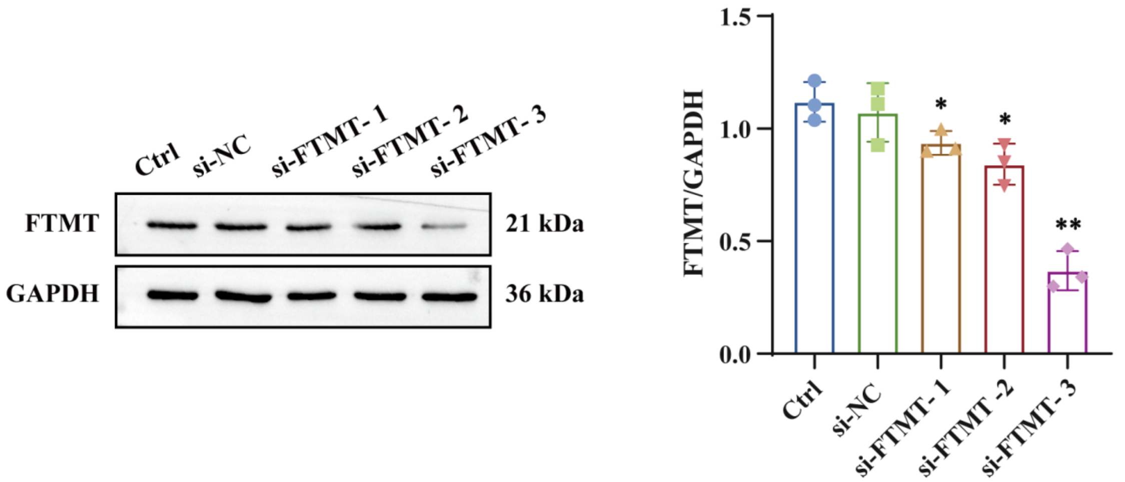

Supplementary Figure S3. VSMCs-specific FTMT gene knockout enhances VSMCs proliferation and migration. Western blot to detect the transfection efficiency of silencing FTMT in VSMCs (n=3). $^{*}P < 0.05$, $^{**}P < 0.01$ vs. siNC group.

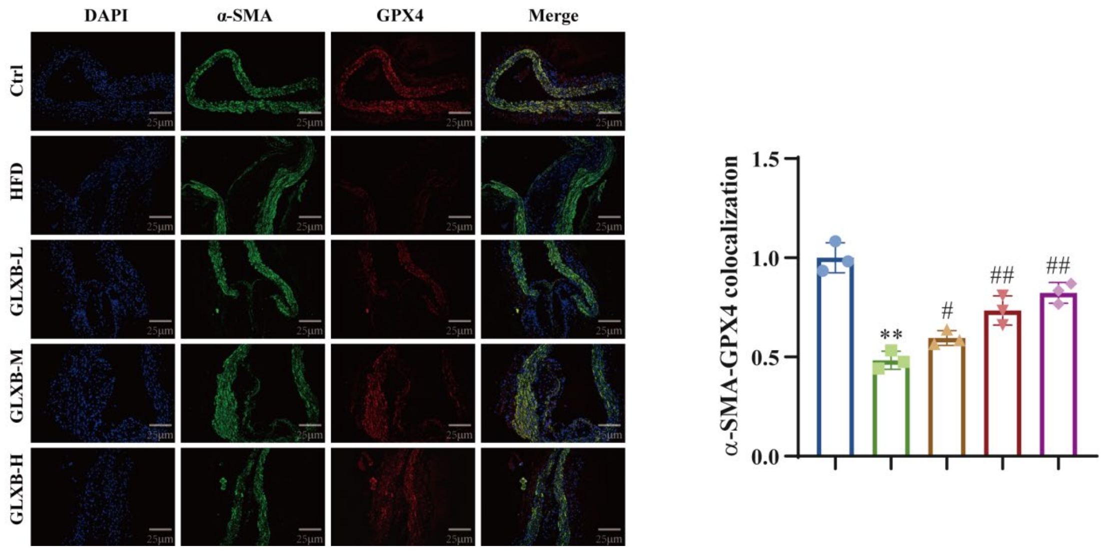

Supplementary Figure S4. GLXB attenuates atherosclerotic plaque formation via NCOA4-dependent suppression of ferroptosis in VSMCs. Immunofluorescence co-localization of α-SMA (green) and GPX4 (red) in aortic VSMCs (n=3, scale bar: 25μm). $^{**}P < 0.01$ vs. Ctrl group, $^{\#}P < 0.05$, $^{\#\#}P < 0.01$ vs. HFD group.

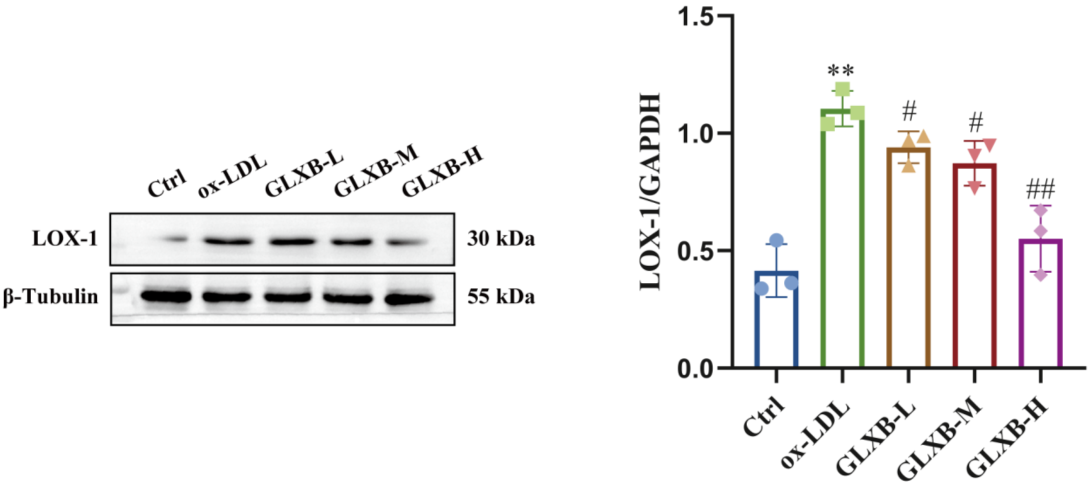

Supplementary Figure S5. The effect of different doses of GLXB on the protein expression of LOX-1. Western blot to detect the effect of different doses of GLXB on LOX-1 protein expression (n=3). $^{**}P < 0.01$ vs. Ctrl group; $^{\#}P < 0.05$, $^{\#\#}P < 0.01$ vs. ox-LDL group.
